# Antigen-Independent Selection of T15 Idiotype During B-Cell Ontogeny In Mice

**DOI:** 10.1155/1991/45352

**Published:** 1991

**Authors:** Meenal Vakil, David E. Briles, John F. Kearney

**Affiliations:** 1 Division of Developmental and Clinical Immunology Department of Microbiology, and The Comprehensive Cancer Center University of Alabama at Birmingham Birmingham Alabama 35294 USA; 2 263 Tumor Institute University of Alabama at Birmingham UAB Station Birmingham Alabama 35294 USA

## Abstract

Precursors of B cells capable of responding to a T-independent form of phosphorylcholine (PC) in splenic focus assays were detected in the spleens of neonatal mice as early as 4 days after birth. The earliest anti-PC B cells were T15^-^. T15^+^ foci-forming B cells were first detected 6 days after birth and expanded rapidly to constitute greater than 80% of the total PC-specific foci by day 10. Injection of heat-killed *S. pneumoniae* (R36A) into neonatal mice resulted in priming of the antibody response to PC, with an idiotype profile reflecting that of precursors of foci-forming B cells at the time of antigen administration. Priming of 2-dayold mice with 2 X10^6^ and 2 X10^7^ R36A induced a five- and ten-fold increase in the antibody response to phosphorylcholine 6 to 8 weeks later. However, only 10 to 15% of the serum antibodies expressed the normally dominant T15 idiotype. Doses below 2 x10^5^ R36A showed no detectable priming activity. PC-specific hybridomas derived from mice injected with 2 X10^7^ R36A 2 days after birth lacked the idiotypic and molecular characteristics typical of T15^+^ antibodies. Antibodies to phosphorylcholine, raised by immunization of 6-week-old mice are normally protective against pneumococcal infection. However, serum antibodies from mice treated with R36A 2 days after birth and responding to phosphorylcholine following challenge with R36A at 6 weeks of age failed to protect against deliberate infection with virulent *S. pneumoniae*. These observations imply that the antigen phosphorylcholine does not play a role in the selective expansion and dominant expression of the T15 idiotype.


*Developmental Immunology*, 1991, Vol. 1, pp. 203–212
Reprints available directly from the publisher
Photocopying permitted by license only

© 1991 Harwood Academic Publishers GmbH
Printed in the United Kingdom

### Antigen-Independent Selection of T15 Idiotype During B-Cell Ontogeny In Mice

MEENAL VAKIL*†, DAVID E. BRILES†, and JOHN F. KEARNEY†

†*Division of Developmental and Clinical Immunology, Department of Microbiology, and The Comprehensive Cancer Center, University of Alabama at Birmingham, Birmingham, Alabama 35294*

Precursors of B cells capable of responding to a T-independent form of phosphorylcholine (PC) in splenic focus assays were detected in the spleens of neonatal mice as early as 4 days after birth. The earliest anti-PC B cells were T15$^-$. T15$^+$ foci-forming B cells were first detected 6 days after birth and expanded rapidly to constitute greater than 80% of the total PC-specific foci by day 10. Injection of heat-killed *S. pneumoniae* (R36A) into neonatal mice resulted in priming of the antibody response to PC, with an idiotype profile reflecting that of precursors of foci-forming B cells at the time of antigen administration. Priming of 2-day-old mice with $2\times10^6$ and $2\times10^7$ R36A induced a five- and ten-fold increase in the antibody response to phosphorylcholine 6 to 8 weeks later. However, only 10 to 15% of the serum antibodies expressed the normally dominant T15 idiotype. Doses below $2\times10^5$ R36A showed no detectable priming activity. PC-specific hybridomas derived from mice injected with $2\times10^7$ R36A 2 days after birth lacked the idiotypic and molecular characteristics typical of T15$^+$ antibodies. Antibodies to phosphorylcholine, raised by immunization of 6-week-old mice are normally protective against pneumococcal infection. However, serum antibodies from mice treated with R36A 2 days after birth and responding to phosphorylcholine following challenge with R36A at 6 weeks of age failed to protect against deliberate infection with virulent *S. pneumoniae*. These observations imply that the antigen phosphorylcholine does not play a role in the selective expansion and dominant expression of the T15 idiotype.

KEYWORDS: B-cell development, idiotypes, antibody protection, idiotypic dominance.

### INTRODUCTION

Humoral immune responses to bacterial-associated antigens in inbred strains of mice are often characterized by the dominant expression of one or a few idiotypes (Sher and Cohn, 1972; Hansberg et al., 1977). The antibody response to phosphorylcholine (PC), which is a major immunogenic determinant expressed by a variety of microorganisms, is a striking example of this phenomenon (Potter and Lieberman, 1970; Claflin et al., 1974). In many inbred strains of mice such as BALB/c and C57BL/6, the majority of antibodies in the primary immune response to PC express the TEPC15 (T15) idiotype (Gearhart et al., 1977; Claflin and Cubberly, 1980). Furthermore, it has been shown that monoclonal antibodies expressing this idiotype provide optimal protection against infection by pathogens expressing PC (Briles et al., 1982). Dominant expression of J606 and J558 idiotypes is likewise observed in the immune responses to bacterial levan and $\alpha$1,3 dextran, respectively (Blomberg et al., 1972; Lieberman et al., 1976; Hansberg et al., 1977) in appropriate responder strains of mice.

In the few cases where this phenomenon has been investigated at a molecular level, idiotypic dominance appears to reflect the clonal expansion of one or a few B cells (Claflin and Cubberley, 1980). Given that B cells have the capacity to generate an extremely diverse repertoire, the recruitment of only a handful of B-cell clones in response to these T-independent antigens is intriguing and suggests developmental regulation of a high order. Environmental antigens have often been suggested as

*Corresponding author. Present address: 263 Tumor Institute, University of Alabama at Birmingham, UAB Station, Birmingham, Alabama 35294.

responsible for the preferential expansion during development of B cells that express high-affinity receptors for antigen, thus leading to idiotype dominance (Etlinger et al., 1982). However, it is clear that even in germfree mice T15+ antibodies dominate the responses to phosphorylcholine (Sigal et al., 1977).

In our previous studies we have characterized the specificities of antibodies in the perinatal B-cell repertoire in BALB/c mice and *in vivo* activities of their antibody products (Vakil and Kearney, 1986). Our studies focused primarily on the development of T15 and J558 idiotypes and led to the hypothesis that autoantiidiotypic B cells and/or their antibody products in perinatal mice play a fundamental role in the selection or expansion of target-idiotype-bearing B cells that normally arise later in ontogeny. This view predicts that antigen is not required for the establishment of dominant clones of B cells and that the role of antigen in clonal expansion is secondary to the effects of autoantiidiotypic B cells or their antibody products during ontogeny. This argument is supported by the observation that even in germfree mice T15 emerges as the dominant idiotype in response to challenge with R36A (Sigal et al., 1977; Etlinger and Heusser, 1986). A corollary to this hypothesis is that premature exposure to antigen will interfere with establishment of clonal dominance.

Here we report a set of experiments that examines the effects of exposure to PC early during B-cell development on the magnitude and characteristics of the anti-PC response upon reexposure to the same antigen as adults, with emphasis on the idiotype profile and protective capacity of anti-PC antibodies. These studies show that extrinsic antigen is not responsible for the dominant expression of T15 idiotype in the response to PC and suggest, instead, a key role for developmental idiotypic regulation of this germline-encoded antibody response.

### RESULTS

### Precursors of PC-Reactive and T15+ B Cells During Ontogeny

It has been previously shown in a functional assay for B-cell precursors involving an organ-fragment culture system that splenic B cells capable of responding to PC could not be detected in mice until 4–5 days after birth (Sigal et al., 1977). We have employed a modification of this assay (Hurwitz et al., 1982) to enumerate splenic B cells in neonatal mice that can respond to a T-independent form of PC (a heat-killed vaccine of *S. pneumoniae* [R36A]) to further assess development of the T15 idiotype as a function of age (Table 1). Until 3 days after birth no detectable splenic B cells responded to PC in fragment cultures. Spleen cells from 4-day-old mice contained precursors of foci producing PC-specific antibodies, but these did not express the T15 idiotype. Precursors capable of developing into T15+ foci did not appear until 6 days of age. The frequencies of T15+ foci increased with age of the donor mice and rapidly outnumbered non-T15 PC-specific foci. By day 10, 80% of the PC-responsive foci expressed the T15 idiotype, which reflects the contribution of

TABLE 1
Number of PC-Specific Foci and Percentages of T15+ Foci as a Function of Development

| Age of donor mice in days[a] | Number of donor mice[b] | Total number of cells analyzed | Number of PC+ foci | Frequency (in $10^6$ spleen cells)[c] | Number of T15+ foci | %T15+ |
|---|---|---|---|---|---|---|
| 2 | 58 | $2\times10^8$ | 0 | 0 | 0 | 0 |
| 3 | 52 | $2\times10^8$ | 0 | 0 | 0 | 0 |
| 4 | 31 | $2\times10^8$ | 1 | 0.125 | 0 | 0 |
| 6 | 17 | $2\times10^8$ | 7 | 0.875 | 2 | 28.5 |
| 7 | 13 | $3\times10^8$ | 15 | 1.2 | 6 | 40.0 |
| 8 | 7 | $10^8$ | 7 | 1.7 | 4 | 57.1 |
| 9 | 5 | $10^8$ | 13 | 3.2 | 9 | 69.2 |
| 10 | 6 | $1.8\times10^8$ | 30 | 4.1 | 24 | 80.0 |
| 12 | 4 | $1.5\times10^8$ | 28 | 4.6 | 25 | 89.2 |
| 21 | 1 | $1.2\times10^8$ | 18 | 3.7 | 16 | 88.8 |
| 42–46 | 1 | $2\times10^8$ | 69 | 8.6 | 63 | 91.3 |

[a]The day of birth was counted as day 1. Thus, an age of 2 indicates 1 day after birth, age of 3 indicates 2 days after birth, etc.
[b]Spleen cells from mice born within 12 hr of each other were pooled. Results from experiments performed on different days were pooled for the table.
[c]Calculated as per Sigal et al. (1977).

T15$^+$ B cells in adult mice. These results suggest that following initial expression of T15$^-$ B cells, T15$^+$ B cells rapidly expand during the second week of neonatal development, leading to the dominant expression of this idiotype later in life.

### Dominance of Non-T15 Antibodies in Mice Prematurely Exposed to PC

Based on the results obtained before, we chose to explore possible mechanisms leading to this idiotypic dominance by investigating the priming capacity of heat-killed R36A to influence the PC response as a function of age. Mice were injected with $2 \times 10^7$ R36A at intervals between 2 to 21 days after birth. A group of control mice was injected with saline at 2 days of age. At 7 weeks of age, all mice were injected with $2 \times 10^8$ R36A, and bled 7 days later. As shown in Fig. 1, in all mice pre-exposed to antigen, the levels of PC-binding IgM antibodies were elevated. Furthermore, greater than 95% of these antibodies expressed a $V_H$ S107 associated idiotype defined by monoclonal antibody TC54 (Desaymard et al., 1984). However, in mice pretreated with R36A at 2 or 4 days after birth, only 10 to 15% of the PC-binding antibodies expressed the T15 idiotype as determined by binding to the monoclonal anti-T15 antibody AB1-2 (Kearney et al., 1983). This antiidiotype antibody reacts with the prototype idiotope expressed on anti-PC antibodies dependent on the combination of $V_H$ S107-encoded heavy chains and $V_K$ 22-encoded light chains. In mice pretreated at 6 days of age, the T15 idiotype constituted 50 to 60% of the total anti-PC response, and in mice pretreated with R36A at days 9, 12, 15 or 21 after birth, greater than 85% of the antibodies were T15 positive, typical of adult values.

These results show that PC-specific B cells in neonatal mice are primed by neonatal exposure to R36A to generate an augmented immune response later in life. Further, the idiotype profile of the neonatally primed anti-PC responses in adults reflects the ratios of non-T15 and T15 foci-forming B cells present at the time of treatment. In addition to treatment with whole R36A, injection of 5 $\mu$g of pneumococcal polysaccharide extracted from capsules of type III pneumococci into 2-day-old mice also resulted in priming of non-T15 anti-PC antibodies (results not shown). These experiments support the hypothesis that during normal development, selective expansion of T15$^+$ B cells by endogenous elements rather than exogeneous antigen leads to the dominant expression of the T15 idiotype in adult mice.

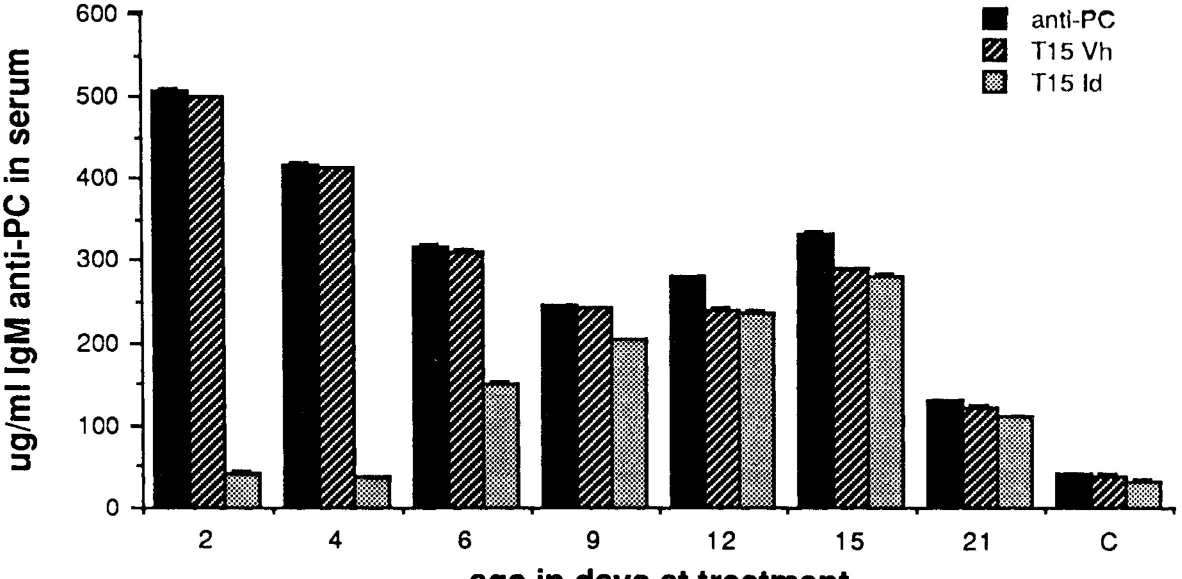

FIGURE 1. Effects of neonatal administration of R36A on the subsequent antibody response to PC. Mice were injected with $2 \times 10^7$ R36A at the ages indicated on the abscissa, rested, and challenged with $2 \times 10^8$ R36A at 7 weeks of age. Immune sera were quantitated as described in Methods. Data indicate levels of anti-PC antibodies (solid bars), T15 $V_H$ expressing antibodies (cross-hatched bars), and T15$^+$ antibodies (stippled bars) in the sera of control and treated mice.

**Relationship of Antigen Dose to Its Priming Capacity**

In mice pretreated with R36A at 2 or 4 days after birth, the concentration of T15-reactive IgM antibodies in the serum after challenge with R36A as adults was comparable to that of control mice. However, T15-positive antibodies constituted only a small portion of the total anti-PC antibodies (Fig. 1). These results suggest that lack of T15 dominance in these mice did not reflect suppressive effects of the antigen on $T15^+$ B cells. To test this hypothesis, the following experiment was conducted. Groups of 2-day-old mice were injected with $2 \times 10^8$ R36A (the optimal immunogenic dose in adult mice), or tenfold dilutions thereof, with $2 \times 10^3$ R36A being the lowest dose tested. Following a rest period of 6 weeks, the mice were challenged with $2 \times 10^8$ R36A.

As shown in Fig. 2, mice pretreated with $2 \times 10^8$ R36A failed to mount a serum-antibody response following challenge with the same dose of antigen as adults, suggesting that this dose had rendered available PC-specific B cells functionally inactive. Neonatal exposure to $2 \times 10^6$ and $2 \times 10^7$ R36A resulted in priming of the non-T15 component in the adult response to PC. $2 \times 10^5$ R36A yielded a small increase over control anti-PC levels, however, $2 \times 10^4$ or lower doses of R36A failed to alter the PC-specific immune response. It is of note that in all mice primed with $2 \times 10^7$ or lower doses of R36A, the level of $T15^+$ IgM antibodies was comparable to that in the group of control mice challenged at the same age and that none of the doses tested resulted in either the induction or suppression of T15 antibodies. Thus, the resulting expansion of the $T15^-$ component in the adult response to PC in neonatally treated mice did not appear to occur at the expense of $T15^+$ B cells. Furthermore, the results of this and the previous experiment showed that priming of non-T15 antibodies in normal mice results only at appropriate times during development.

**Characteristics of the Adult Antibody Response in Mice treated neonatally with PC**

Hybridomas were generated from normal control mice and from mice treated with $2 \times 10^7$ R36A 2 days after birth after challenge with $2 \times 10^8$ pneumococci at 7 weeks of age to examine in detail the molecular characteristics of $T15^-$ antibodies generated in the latter group. Monoclonal antibodies produced by these hybridomas were analyzed for reactivity with PC-BSA and with a panel of anti-$V_H$ and anti-idiotypic antibodies (described in Cerny et al., 1982;

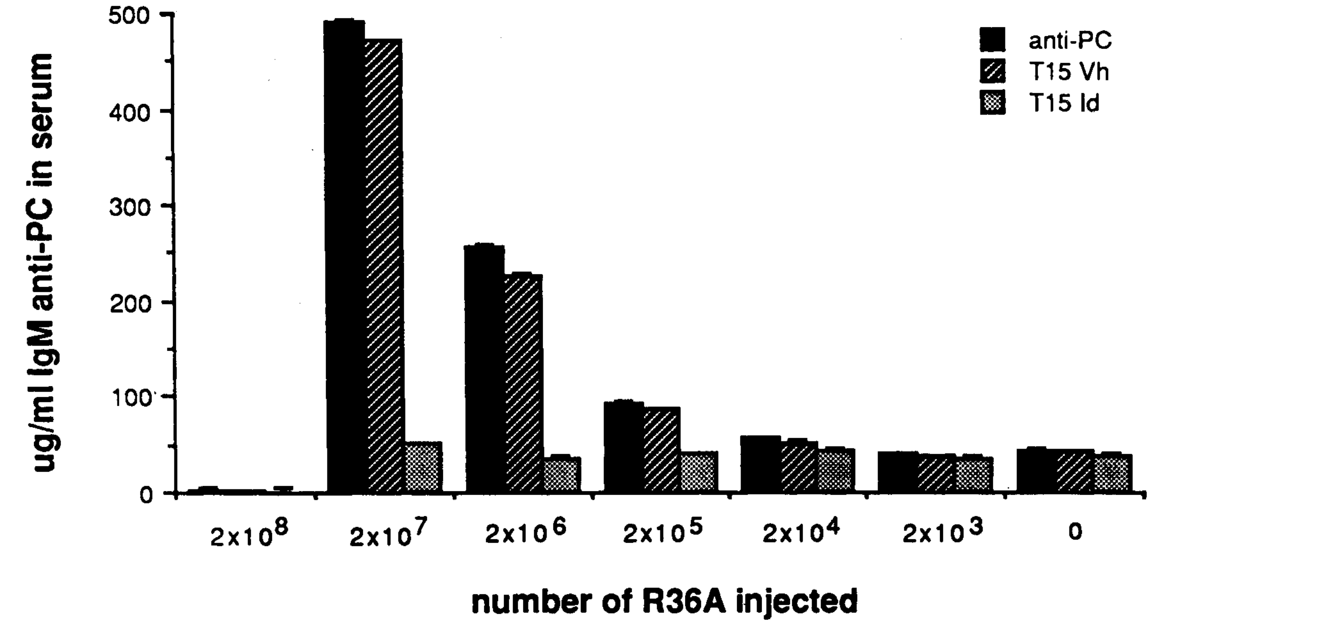

FIGURE 2. Effects of neonatal administration of different doses of R36A on the subsequent antibody response to PC. Mice were injected 2 days after birth with doses of R36A indicated on the abscissa, rested, and challenged with $2 \times 10^8$ R36A at 7 weeks of age. Immune sera were quantitated as described in Methods. Data indicate levels of anti-PC antibodies (solid bars), T15 $V_H$-expressing antibodies (cross-hatched bars), and $T15^+$ antibodies (stippled bars) in the sera of control and treated mice.

Kearney et al., 1983). As expected, seven out of seven monoclonal antibodies derived from normal untreated mice react with PC-BSA, TC-54, AB1-2, GB4-10, and MAID5-4 (Table 2), as does the prototype T15$^+$ antibody BH8 previously generated in our laboratory (Pollok et al., 1984). However, only one of nine monoclonal antibodies derived from mice neonatally injected with R36A showed PC binding associated with the characteristic T15-idiotype profile. The other eight hybridoma antibodies reacted with PC-BSA and the $V_H$ S107-specific antibody TC54 but not with the T15-specific antiidiotype antibodies and were categorized as T15$^-$.

Dot blot analyses of total cytoplasmic RNA using $V_H$ S107 as well as $V_K$22-, $V_K$8- and $V_K$ 24-specific probes suggested that while all of the hybridomas generated from normal untreated mice expressed productively rearranged $V_H$ S107 as well as $V_K$ 22 as does the prototype hybridoma BH8, only the singular T15-idiotype positive hybridoma from the R36A-treated mice utilized this combination of $V_H$ and $V_K$ genes. RNA from the remaining eight hybridomas from the latter group hybridized with the $V_H$ S107 probe consistent with the reactivity of their antibody products with TC54. However, six of these hybridoma expressed $V_K$ 24-encoded light chains characteristic of M167-idiotype-bearing antibodies and one hybridoma expressed light chains encoded by $V_K$ 8 genes characteristic of M603 antibodies. The light-chain gene used by the remaining hybridoma could not be determined.

**Failure to Induce Protective Immunity in Sera of Mice Neonatally Exposed to R36A**

Anti-PC antibodies expressing the T15 rather than non-T15 idiotypes have been previously shown to be much more protective against virulent *S. pneumoniae* (Briles et al., 1982). We therefore sought to test whether the anti-PC antibodies generated in adult mice that had been primed as neonates with small doses of R36A would protect against infection by the virulent strain WU2. Sera were collected and pooled from mice that had been injected with R36A 2 days after birth and again at 7 weeks of age and that expressed the lowest content of T15 antibodies, or from control mice, following challenge as adults as described (Fig. 1) and were transferred into groups of recipient CBA/N×DBA/2 F1. male XID mice. Sera were diluted in saline to contain approximately 30 $\mu$g of IgM anti-PC antibody in 0.2 ml and injected intraperitoneally in recipient XID mice. One hour later, these mice were injected intravenously with 100 live *S. pneumoniae* (WU2) and mortality was measured over a period of 7 days.

As shown in Table 3, all mice in the control group injected with WU2 alone or with a dilution of serum from normal unimmunized mice comparable to that of the immune serum pool died with 3 days. Five of seven mice that received immune sera from normal adult mice challenged within R36A survived the length of the experiment. However, all four mice that received sera from neonatally immunized mice that had been treated with R36A died within the

TABLE 2
Idiotypic Characteristics and $V_H/V_L$ Utilization of PC-Specific Hybridomas from Control and Neonatally PC-Treated Mice

| | Hybridomas reactivity towards | | | | | RNA hybridization to | | | |
|---|---|---|---|---|---|---|---|---|---|
| | PC | TC54 | MAID5.4 | AB1-2 | GB4-10 | $V_H$ S107 | $V_K$ 22 | $V_K$ 24 | $V_K$ 8 |
| C1 | ++ | ++ | ++ | ++ | ++ | + | + | − | − |
| C2 | ++ | ++ | ++ | ++ | ++ | + | + | − | − |
| C3 | ++ | ++ | ++ | ++ | ++ | + | + | − | − |
| C4 | ++ | ++ | ++ | ++ | ++ | + | + | − | − |
| C5 | ++ | ++ | ++ | ++ | ++ | + | + | − | − |
| C6 | ++ | ++ | ++ | ++ | ++ | + | + | − | − |
| C7 | ++ | ++ | ++ | ++ | ++ | + | + | − | − |
| R1 | ++ | ++ | ++ | ++ | ++ | + | + | − | − |
| R2 | ++ | ++ | − | − | − | + | − | + | − |
| R3 | ++ | ++ | − | − | − | + | − | + | − |
| R4 | ++ | ++ | − | − | − | + | − | + | − |
| R5 | ++ | ++ | − | − | − | + | − | + | − |
| R6 | ++ | ++ | − | − | − | + | − | + | − |
| R7 | ++ | ++ | − | − | − | + | − | + | − |
| R8 | ++ | ++ | − | − | − | + | − | − | + |
| R9 | ++ | ++ | − | − | − | + | − | − | − |

first 3 days. These results show that neonatal (premature) exposure to R36A does not induce protective immunity even though high titers of IgM anti-PC antibodies are produced in such mice following exposure to R36A as adults.

TABLE 3

| Treatment of serum donor | | Alive/dead postchallenge | |
|---|---|---|---|
| neonatal | adult | 3 days | 10 days |
| None | R36A | 7/0 | 5/2 |
| R36A | R36A | 0/4[a] | 0/4[c] |
| None | None | 0/7[b] | 0/7[a] |
| No serum | | 0/7[b] | 0/7[a] |

[a]Different from none/R36A at $p < 0.01$.

[b]Different from none/R36A at $p < 0.001$.

[c]Different from none/R36A at $p < 0.06$.

### DISCUSSION

The phenomenon of B-cell clonal dominance in immune responses to several exogenous antigens has been repeatedly observed in inbred strains of mice. The mechanisms by which dominant idiotypes are selected are not well understood. Three hypotheses have emerged to explain this phenomenon. The first, derived from analysis of the PC response suggested a role for environmental antigen in selective expansion of antigen-specific B-cell clones (Etlinger et al, 1982). Later, Klinman and Stone (1983) observed that the T15 idiotype was dominant in bone-marrow-derived surface immunoglobulin negative pre-B cells capable of differentiating into antibody-secreting foci in organ cultures. They therefore proposed that dominance of the T15 idiotype was regulated at the level of DNA rearrangements and was the result of a biased association of $V_H$ S107 with $V_K$ 22 in PC-specific antibodies (Klinman and Stone 1983). Finally, based on our studies on the functional characteristics of monoclonal antiidiotypic antibodies derived from perinatal B-cell hybridomas (Vakil and Kearney, 1986; Vakil et al., 1986), we proposed that dominant expression of the T15 idiotype results from selective expansion of B cells expressing this idiotype by autoantiidiotypic B cells during ontogeny, as had been suggested previously by Köhler (1979).

It has been demonstrated that in mice lethally irradiated and reconstituted with normal adult bone marrow, the T15 idiotype fails to dominate the antibody response to PC (Kaplan et al., 1978; Augustin et al., 1977; Wemhoff and Quintans, 1987) despite the fact that bone-marrow-transplanted mice are exposed to an environment similar to normal conventionally reared mice. Furthermore, molecular mechanisms operating at the level of DNA rearrangements in the donor hemopoietic tissue in marrow-transplant recipients are not likely to be distinct from those operating in normal mice. Thus, the first two hypotheses stated before are unable to explain the loss of T15 dominance in lethally irradiated bone-marrow-transplanted mice. It appears therefore that although exposure to antigen may aid in the expansion of dominant idiotypes, it is not the limiting factor in the establishment of dominant B-cell clones.

We have previously reported the stimulatory effects of the anti-T15 antibody BD2 and the antianti-T15 antibody DB3, products of perinatal B-cell hybridomas (Vakil and Kearney, 1986). Experiments described in the current report establish that the antigen PC does not play a significant role in the initial expansion of $T15^+$ B cells during ontogeny. We have assessed (i) the functional status and idiotypic characteristics of PC-responsive precursors in neonatal mice and (ii) the effects of exposure of newborn mice to small amounts of antigen on subsequent immune response to PC as adults.

The frequencies of $T15^+$ B cells as a function of age, directly assayed by immunofluorescent staining of IgM B cells by anti-T15 antibodies AB1-2 and GB4-10, have been reported (Bast et al., 1984; Pollok and Kearney, 1984). Here we demonstrate that precursors of PC-responsive B cells are present in the spleens of 4-day-old BALB/c mice and can develop into antibody-forming foci in fragment cultures. However, the responding B cells do not express the T15 idiotype. PC-responsive $T15^+$ B cells begin to appear around 6 days after birth when they constitute about 29% of the PC-reactive precursors and they increase in numbers nearing adult proportions of T15 idiotype by day 10. Similar results have been reported by Fung and Köhler (1980) and Teale and Kearney (1986).

To permit detection of minor changes in T15 expression as a function of age, we pooled donor spleen cells only from mice born within 18 hours of each other. The trend of increase in percent $T15^+$ foci between days 4 and 42 calculated using a rank test is statistically significant with $p < 0.001$. Sigal and Klinman have previously conducted similar experiments using PC-Hemocyanin as the stimulating antigen (Sigal et al., 1977), and reported that PC-responsive precursors were detected at a frequency of 0.87 per $10^6$ cells in spleens of 4–5-day-old (BALB/c ×C57BL/6)F1 mice and that these lacked the T15 idiotype. However, when spleen cells pooled from 6–8-day-old, 8–10-day-old, or adult mice were analyzed in similar assays, $T15^+$ B cells were found to dominate in the population of PC-responsive B cells. Because of the rapid emergence of the T15 idiotype, the detection of non-T15 PC-specific precursors only at very low frequencies in spleens of 3- or 4-day-old donor mice precluded further analysis. Our results confirm that $T15^-$ B cells are functional before $T15^+$ B cells during ontogeny, however, the latter are selectively expanded and rapidly outnumber the former, leading to the dominance of the T15 idiotype later in life.

The relative appearance of functional $T15^-$ and $T15^+$ PC-reactive B cells during ontogeny is also reflected by their response to antigen priming during the first two weeks of life. Antigen exposure up to 4 days after birth resulted in the priming of $T15^-$ antibodies, whereas exposure at 6 days and beyond primed both $T15^-$ and $T15^+$ B cells. The content of T15 idiotype in the subsequent response to PC reflects closely the proportion of $T15^+$ B cells within the PC-reactive B-cell pool at the time of priming. These observations suggest that there is selective and rapid expansion of $T15^+$ B cells in neonatal mice between 6 and 10 days of life. To determine whether antigen plays a role in the events that lead to the dominant expression of the T15 idiotype, we analyzed the priming capacity of various doses of R36A in 2-day-old mice. Three findings were of note. Firstly, the optimal adult dose of $2 \times 10^8$ bacteria appeared to induce tolerance to PC when injected into 2-day-old mice. Secondly, in mice pretreated with doses of R36A lower than the optimal adult dose, there was no obvious impairment of the $T15^+$ component of the anti-PC response at 7 weeks of age. Thirdly, it is apparent that doses of R36A smaller than $2 \times 10^5$ fail to produce any effect that is readily evident in a subsequent immune response to PC.

Although we have not conducted experiments to determine whether the PC-reactive cells have been physically deleted in mice neonatally injected with $2 \times 10^8$ R36A, impairment of the anti-PC response is obvious. Considering that $T15^-$ B cells may be the only PC-responsive B cells present at this age, it is not clear why this treatment also affects the $T15^+$ response since B cells expressing this idiotype do not become functional until about 6 days after birth. However, it is probable that with high doses of R36A, antigen may persist in the neonatal mice at a high concentration long enough to inactivate emerging $T15^+$ B cells. Alternately, the functional elimination of $T15^-$ B cells may interfere with idiotypic interactions, which we propose are responsible for the development of functionally competent $T15^+$ B cells (Vakil and Kearney, 1991). Injection of $2 \times 10^7$ or lower doses of R36A into neonatal mice does not alter T15 expression, suggesting that $T15^+$ B cells may be absent in 2–4-day-old mice, or if present, they are functionally incompetent and unable to respond to the antigen. The failure of neonatal injection of small doses of R36A ($< 2 \times 10^5$ organisms) to alter the levels of anti-PC antibodies or T15 expression is consistent with the observation that the T15 idiotype is invariably dominant in mice raised under germfree or conventional environments (Sigal et al., 1977; Etlinger and Heusser, 1986), despite the ubiquitous nature of PC antigen in the latter (Potter, 1977). Furthermore, in lethally irradiated, bone-marrow-transplanted mice, the absence of T15 idiotype suggests that small amounts of environmental PC are not sufficient for the selective expansion of $T15^+$ B cells.

We have examined the characteristics of the PC-reactive component of the immune response that is primed by early exposure to R36A. Hybridomas generated from mice neonatally exposed to R36A showed that the majority of hybridizing B cells produced anti-PC antibodies that utilize the $V_H$ S107 heavy-chain gene, however, it is in combination with $V_K24$ rather than $V_K$ 22. Therefore, these antibodies did not arise from $T15^+$ B cells by somatic mutation, nor do they belong to the group II or group III anti-PC antibodies described by Chang et al. (1984). These non-T15 antibodies also failed to confer protective immunity in recipient XID mice against virulent *S. pneumoniae.* We have further reported in a separate study that mice primed with R36A 2 days after birth also fail to respond to a structurally distinct but idiotypically linked antigen $\alpha$1,3 dextran (Vakil and Kearney, 1991). Therefore, our results suggest that premature antigen priming may lead to dominance by clones secreting antibodies that do not protect against infection, and as well, distort immune responses to unrelated antigens. These considerations may be relevant to present attempts to develop and use immunogenic polysaccharide vaccines in human infants.

We have previously reported the generation of autoantiidiotypic B cell hybridomas from unmanipulated fetal and newborn mice. Passive transfer of the purified IgM anti-T15 antibody BD2 into newborn mice resulted in an enhancement of the immune response to PC in these mice as adults. In fact, treatment with BD2 on days 5 and 7 induced maximal enhancement of response to PC with dominant expression of T15 idiotype; treatment on day 10 induced suboptimal enhancement; and treatments beyond day 10 produced minimal or no enhancement (Vakil and Kearney, 1986). Thus, the age window during which antibody BD2 induces maximal enhancement of T15 coincides with the age at which spleen cells from donor mice normally acquire the capacity to generate $T15^+$ foci in fragment cultures. It also corresponds with the developmental stage when $T15^+$ B cells can be primed by R36A to give an enhanced response to PC later in life. The studies presented here support our hypothesis that idiotypic dominance of T15 results from the selective expansion of particular B cells and that endogenous BD2-like antiidiotypic B cells and/or their antibody products may be involved in the establishment of $T15^+$ B cells in young unimmunized mice.

The mechanisms involved in the proposed B-cell interactions are not obvious. We have observed that development of anti-T15 B cells precedes that of $T15^+$ B cells (Pollok and Kearney, 1984). Similarly, anti-T15 antibodies that were proposed to regulate the expression of T15 idiotype *in vivo* have been observed in serum and cultures of spleen cells from 4–7-day-old mice (Strayer and Köhler, 1976). Perhaps, during ontogeny, antiidiotypic B cells deliver requisite signals that select newly emergent target-idiotype-bearing B cells, which then acquire the capacity to respond to antigen. According to this hypothesis, $T15^+$ B cells are generated throughout life as a result of appropriate rearrangements at the heavy- and light-chain loci in newly formed B cells. However, in the event that these B cells are not selected by the appropriate anti-T15 B cell(s), they will eventually be eliminated. In this context, we have previously shown that when appropriate autoantiidiotypic B cells in newborn mice are inactivated, development of later-appearing $T15^+$ as well as $J558^+$ B cells is abolished (Vakil et al., 1986). The observation that antibody BD2 induced maximal enhancement of T15 only between days 5 and 7 suggests that this time window is critical for the idiotypic interactions between anti-T15 and T15 B cells. In fact, injection of purified monoclonal antibodies of either T15 idiotype or of the connected J558 idiotype during this time also interfered with the development of endogenous $T15^+$ B cells, presumably by blocking the interaction between anti-T15 and T15 B cells (Vakil and Kearney, 1991). These observations suggest that autoantiidiotypic B cells are obligatory components of idiotypic cascades responsible for the establishment of idiotype dominance.

Together, these observations suggest that dominance of the T15 idiotype and, therefore, the development of protective immunity to PC-expressing pathogens is not directed by antigen. Rather, antigen administration to adults results in the selective expansion of $T15^+$ B cells initially selected and expanded by endogenous antiidiotypic antibodies during development.

This phenomenon is not restricted to the anti-PC response. Similar experiments have been conducted by injection of suboptimal doses of the T-independent antigen $\alpha$1,3 dextran into newborn mice to investigate the phenomenon of idiotype dominance of the J558. When such treatments were followed by antigen challenge at 6 to 7 weeks of age, we likewise observed a skewing of the immune response to an overproduction of antibodies lacking the normally dominant J558 idiotype (Vakil and Kearney, 1991). Therefore, the dominant expression of one or a few idiotypes commonly observed in immune responses to bacterial antigens does not result from environmental priming during development. Rather, we suggest that it may be the result of regulatory B-cell circuits within the immune system that have evolved to provide an optimal first line of defense toward infectious agents in the form of restricted sets of optimally protective antibodies.

### METHODS

### Mice

Male and female BALB/c mice and female CBA/N and male DBA/2 mice were obtained from Jackson Laboratories, Bar Harbor, ME, and bred in our facility to produce BALB/c and (CBA/N ×DBA/2)F1 offspring used in these studies.

### Splenic Focus Assays

The splenic focus culture system modified by Hurwitz et al. (1982) was employed to quantitate the frequency of PC and T15-positive B-cell precursors. $2 \times 10^7$ spleen cells from neonatal (2–46 days old) BALB/c mice were transferred i.v. to each X-irradiated syngeneic recipient. This number of donor cells resulted in a frequency always <0.11 PC-specific foci per fragment, thereby ensuring the clonal nature of the assay (a frequency of ≤0.37 positive foci per fragment is required for clonality according to Poisson distribution). Recipient spleens were removed 24 hr after transfer, diced to approximately 1-mm cubic fragments, and individually placed *in vitro* with $5 \times 10^6$ heat-killed R36A cells/ml supplemented to the tissue-culture medium. Fragments were fed (without antigen) on days 3, 7, 11, and 15 of culture and the supernatants screened for PC-binding and AB1-2 idiotope-bearing antibody by solid-phase ELISA on days 7, 11, 15, and 21 of culture.

### Immunizations

Neonatal and adult mice were injected intraperitoneally with a heat-killed preparation of *S. pneumoniae* strain R36A in saline. Adult mice were bled 1 week after immunization from the periorbital sinus and the sera were stored frozen.

### Quantitation of Serum Antibody

PC-specific antibodies in the sera of immunized mice were analyzed in a quantitative ELISA as previously described (Stohrer and Kearney, 1984). Briefly, polyvinyl microtiter plates were coated with PC-BSA or monoclonal anti-Id antibodies, blocked and incubated with serial dilutions of serum samples from individual mice, or known amounts of monoclonal standards. The assays were developed with phosphatase-labeled goat antimouse IgM antibodies and phosphatase substrate. The absorbance values were read in a Titertek multiscan (Flow Laboratories) and the standard equivalents were calculated based on reference O.D. values of monoclonal standards.

### Hybridoma Construction and Screening

Hybridomas were constructed from R36A-immunized mice by fusion with the nonsecreting plasmacytoma P3X63.Ag8.653 using standard procedures (Kearney et al., 1979). Hybridoma supernatants were screened in a colorimetric ELISA for binding to ligands described in the text. Briefly, polyvinyl microtiter plates were coated with ligand at a concentration of 2 $\mu$g/ml and blocked with 1% BSA in PBS. Test supernatants were loaded into blocked plates and following overnight incubation were developed with alkaline phosphatase labeled goat antimouse IgM antibodies (Southern Biotechnology Associates, Inc., Birmingham, AL) followed by phosphatase substrate.

Expression of $V_H$ S107 and $V_K$ 22, 24, or 8 was analyzed in dot blots as previously described (White and Bancroft, 1982). Probes specific for $V_H$ S107 and $V_K$ 22, $V_K$ 24, and $V_K8$ were generous gifts of R. Perlmutter and P. Gearhart. Hybridomas BH8 ($V_K$ 22), HPCM27 ($V_K$ 24), and HPCM25 ($V_K$ 8) and the fusion plasmacytoma P3X63.Ag8.653 were used as controls.

### Mouse Protection Assay with Passively Administered Serum Antibodies

CBA/N×DBA/2 F1 male mice were injected intraperitoneally with 0.2 ml control serum or immune serum diluted in PBS to contain 30 $\mu$g of IgM anti-PC antibodies 1 to 2 hr before the intravenous injection of 100 colony-forming units of *S. pneumoniae* strain WU2. Mice were observed for 10 days and deaths were recorded at 24-hr intervals. The concentration of control serum injected was equivalent to the dilution of immune serum containing 30 $\mu$g of antibody in 0.2 ml. Statistical comparisons were made between mice protected with anti-R36A immune sera and mice in other treatment or control groups, using a Cochran-corrected chi-square analysis (Zar, 1984).

### ACKNOWLEDGMENTS

We wish to thank Ann Brookshire for her expertise and patience in preparation of this manuscript. This work has been supported by grants CA 13148, AI30879, AI 14782, AI 21548, and AI 13557, awarded by the National Institutes of Health.

(*Received October* 16, 1990)

(*Accepted December* 20, 1990)

### REFERENCES

Augustin A.A., Julius N.H., and Cosenza H. (1977). Changes in the idiotypic pattern of an immune response, following syngeneic haemopoietic reconstitution of lethally irradiated mice. *In:* The Immune System: Genetics and Regulation,

Sercarz, E., Herzenberg, L.A. and Fox, C. Eds. (New York: Academic Press), pp. 195–199.

Bast B.J.E.G., Cooper M.D., and Kearney J.F. (1984). Cellular expression of idiotopes defined by monoclonal antibodies. Eur. J. Immunol. **14:** 623–628.

Blomberg B., Geckeler W., and Weigert M. (1972). Genetics of the antibody response to dextran in mice. (1972). Genetics of the antibody response to dextran in mice. Science **177:** 178–180.

Briles D., Forman C., Hudak S., and Claflin J.L. (1982). Anti-phosphorylcholine antibodies of the T15 idiotype are optimally protective against *Streptococcus pneumoniae.* J. Exp. Med. **156:** 1177–1185.

Cerny J., Wallich R., and Hämmerling G.J. (1982). Analysis of T15 idiotypic expression on phosphorylcholine specific lymphocytes from individual inbred mice. J. Imunol. **128:** 1885–1891.

Chang S.P., Perlmutter R.M., Brown M.C.K., Huesser C.H., Hood L., and Rittenberg M.B. (1984). Immunologic memory to phosphocholine. IV. Hybridomas representative of group 1 (T15-like) and group II (non-T15-like) antibodies utilize distinct $V_H$ genes. J. Immunol. **132:** 1550–1560.

Claflin J.L., and Cubberley M. (1980). Clonal nature of the immune response to phosphocholine. VII. Evidence throughout inbred mice for molecular similarities among antibodies bearing the T15 idiotypes. J. Immunol. **125:** 551–558.

Claflin J.L., Lieberman R., and Davie J.M. (1974). Clonal nature of the immune response to phosphorylcholine. II. Idiotypic specificity and binding characteristics of anti-phosphorylcholine antibodies. J. Immunol. **112:** 1747–1756.

Desaymard C., Guisti A.M., and Scharff M.D. (1984). Rat anti-T15 monoclonal antibodies with specificity for $V_H$- and $V_H$-$V_L$ epitopes. Mol. Immunol. **21:** 961–967.

Etlinger H.M., Julius M.H., and Heusser C.H. (1982). Mechanism of clonal dominance in the murine anti-phosphorylcholine response. I. Relation between antibody avidity and clonal dominance. J. Immunol. **128:** 1685–1691.

Etlinger H.M., and Heusser C.H. (1986). T15 dominance in BALB/c mice is not controlled by environmental factors. J. Immunol. **136:** 1988–1991.

Fung J., and Köhler H. (1980). Late clonal selection and expansion of the TEPC-15 germline specificity. J. Immunol. **125:** 640–646.

Gearhart P.J., Sigal N.H., and Klinman N.R. (1977). The monoclonal antiphosphorylcholine antibody response in several murine strains: Genetic implications of a diverse repertoire. J. Exp. Med. **145:** 876–891.

Hansberg D., Briles D.E., and Davie J.M. (1977). Analysis of the diversity of the murine response to dextran B1355. II. Demonstration of multiple idiotypes with variable expression in several strains. J. Immunol. **119:** 1406–1412.

Hurwitz J., Tagart V., Schweitzer P., and Cebra J. (1982). Patterns of isotype expression by B cell clones responding to thymus dependent and thymus independent antigens *in vitro.* Eur. J. Immunol. **12:** 342–348.

Kaplan D.R., Quintans J., and Köhler H. (1978). Clonal dominance: Loss and restoration in adoptive transfer. Proc. Natl. Acad. Sci. USA **75:** 1967–1970.

Kearney J.F., Pollok B., and Stohrer R. (1983). Analysis of idiotypic heterogeneity in the anti-$\alpha\rightarrow3$ dextran and anti-phosphorylcholine responses using monoclonal anti-idiotype antibodies. Annals of N.Y. Acad. Sci. **418:** 151–170.

Kearney J.F., Radbruch A., Liesegang B., and Rajewsky K. (1979). A new mouse myeloma cell line which has lost immunoglobulin expression but permits the construction of antibody secreting hybrid cell lines. J. Immunol. **123:** 1548–1550.

Klinman N.R., and Stone M.R. (1983). Role of variable region gene expression and environmental selection in determining the anti-phosphorylcholine B cell repertoire. J. Exp. Med. **158:** 1948–1961.

Köhler H. (1979). Complementary idiotype interaction in the clonal dominance of the anti-PC response. *In:* B Lymphocytes in the Immune Response, Cooper M.D., Mosier, D., Scher, I., and Vitetta, E.S., Eds. (New York: Elsevier North-Holland), pp. 209–216.

Lieberman R., Potter M., Humphrey W. Jr., and Chien C.C. (1976). Idiotypes of inulin-binding antibodies and myeloma proteins controlled by genes linked to allotype locus of the mouse. J. Immunol. **117:** 2105–2111.

Pollok B.A., and Kearney J.F. (1984). Identification and characterization of an apparent germline set of anti-idiotypic regulatory B lymphocytes. J. Immunol. **132:** 114–121.

Pollok B.A., Kearney J.F., Vakil M., and Perry R.P. (1984). A biological consequence of variation in the site of D-$J_H$ gene rearrangement. Nature **311:** 376–379.

Potter M. (1977). Antigen-binding myeloma proteins of mice. Adv. Immunol. **25:** 141–211.

Potter M., and Liberman R. (1970). Common individual antigenic determinants in five of eight BALB/c IgA myeloma proteins that bind phosphorylcholine. J. Exp Med. **132:** 737–751.

Sher A., and Cohn M. (1972). Inheritance of an idiotype associated with the immune response of inbred mice to phosphorylcholine. Eur. J. Immunol. **2:** 319–326.

Sigal N.H., Pickard A.R., Metcalf E.S., Gearhart P.J., and Klinman N.R. (1977). Expression of phosphorylcholine-specific B cells during murine development. J. Exp. Med. **146:** 933–948.

Stohrer R., and Kearney J.F. (1984). Ontogeny of B cell precursors responding to $\alpha$1-dextran in BALB/c mice. J. Immunol. **133:** 2323–2326.

Strayer D.S., and Köhler H. (1976). Immune response to phosphorylcholine. II. Natural "auto"-anti-receptor antibody in neonatal BALB/c mice. Cell. Immunol. **25:** 294–301.

Teale J.M., and Kearney J.F. (1986). Clonotypic analysis of the fetal B cell repertoire: Evidence for an early and predominant expression of idiotypes associated with the $V_H$ 36-60 family. J. Mol. Cell Immunol. **2:** 283–292.

Vakil M., and Kearney J.F. (1986). Functional characteristics of monoclonal auto-anti-idiotype antibodies isolated from the early B cell repertoire of BALB/c mice. Eur. J. Immunol. **16:** 1151–1158.

Vakil M., and Kearney J.F. (1991). Functional relationship between T15 and J558 idiotypes in BALB/c mice. Develop. Immunol. **1:** 213–224

Vakil M., Sauter H., Paige C., and Kearney J.F. (1986). *In vivo* suppression of perinatal multispecific B cells results in a distortion of the adult B cell repertoire. Eur. J. Immunol. **16:** 1159–1165.

Wemhoff G.A., and Quintans J. (1987). Alterations of idiotypic profiles: The cellular basis of T15 dominance in BALB/c micel J. Mol. Cell. Immunol. **3:** 307–320.

White B.A., and Bancroft F.C. (1982). Cytoplasmic dot hybridization. Simple analysis of relative mRNA levels in mutliple small cell or tissue samples. J. Biol. Chem. **257:** 8569–8572.

Zar J.H. (1984). Biostatistical Analysis (Englewood Cliffs, NJ: Prentice-Hall), p. 178.

